# Supplementary material for: Learning and Developing Together for Improving the Quality of Care in a Nursing Home, Is Appreciative Inquiry the Key?
Source: Healthcare (Basel). 2023 Jun 24;11(13):1840. doi: 10.3390/healthcare11131840 (PMC10340465; doi:10.3390/healthcare11131840)
Supplement: Supplementary file 1 [file healthcare-11-01840-s001.zip › healthcare-2387977-supplementary.pdf]

Supplementary Material A: Interview protocol evaluation appreciative inquiry

Introduction

• Welcome someone and thank you for the time of the interview

• Indicate the purpose of the interview: appreciatively changing experiences of retrieval of the sessions

• Indicate approximately how long the interview lasts: 10-15 minutes

• Tell that it is recorded, literally elaborated, the content is put back for reading and then analyzed and included in the data collection and descriptions of the research project Care teams in their power.

Interview questions:

1. Did you participate in the team sessions? 1, 2 or 3x?

2. How do you look back on the sessions? Or if someone has not been: have you heard from others about the sessions and if so what?

3. What did you like most about the sessions? What has stuck with you the most and why? What have you wondered about and why?

4. What did you take away from it? What do you apply in your work now? What has it brought you as a person, to your team and the location?

5. What does it take to keep it alive?

6. Focused on the goals:

   a. Have you felt heard in the sessions? And how is this now?
   b. Do you feel that you are now involved in changes?
   c. Do you feel free to express your opinion?
   d. Did you get to know each other better?
   e. Has the atmosphere and cooperation changed and if so how?
   f. Have the sessions made it easier to talk openly with each other about (difficult) topics?
   g. Did the sessions motivate us to come to change together in order to take even better care of the residents?

Completion

• Ask if someone has been able to say everything they wanted to say; does anyone want to add anything?

• Thank someone for the contribution

• Ask if there are any questions about the editing of the recording

Supplementary Material B: Instructions appreciative interviews Discovery

You are about to ask a colleague about her work. Central to this conversation is the positive theme of this afternoon, namely

`We provide the best care for our residents together!`

We are looking for positive - proud - stories about working in healthcare at this location. You both must have great stories to tell. Be curious about your colleague's story. Ask her what makes a situation special for her/him, what she/he considers important in her/his work.

You have 20 minutes for the interview. Then your colleague will interview you for 20 minutes.

Below are 3 questions for the interview. Ask the other person which question they want to start with and why they start with this question. Make sure that all questions are covered in these 20 minutes.

Take notes on this paper during the conversation so that you can recount it later in the large group. Include striking, surprising comments and/or reasons that make it a story to be proud of.

For example: a description of 'good care', conditions for providing good care and aspects that are important/valuable to the interviewee.

Appreciative questions:

Describe a situation in which you felt you provided very good care.<br>• Tell me what happened: what did you see, do, feel?<br>• Who were involved?<br>• What did the other(s) do?<br>• What ensured that good care was provided?	
Describe a situation in which you enjoyed working with a colleague.<br>• Tell me what happened: what did you see, do, feel?<br>• Who were involved?<br>• What did the other(s) do?<br>• What ensured that good care was provided?	
Describe a situation in which you worked well with a resident's family.<br>• Tell me what happened: what did you see, do, feel?<br>• Who were involved?<br>• What did the other(s) do?<br>• What ensured that good care was provided?	

Supplementary Material C: Questionnaire learning climate

	These 16 statements relate to the learning climate in the team.<br>You can indicate per statement to what extent you think this occurs in your team.<br>D = goals<br>O = development<br>T = teamwork<br>V = safety<br>C= communication						
		In my team:	Never	Some times	Regularly	Often	Always
1	D	we have the same goal.	☐	☐	☐	☐	☐
2	D	there is a high degree of agreement on what needs to be achieved.	☐	☐	☐	☐	☐
3	D	Do all team members feel committed to the goals set?	☐	☐	☐	☐	☐
4	T	there is a willingness to take on less pleasant things.	☐	☐	☐	☐	☐
5	T	there is respect for a different opinion of colleagues.	☐	☐	☐	☐	☐
6	T	there is appreciation for the skills and talents of another.	☐	☐	☐	☐	☐
7	V	may existing principles be called into question.	☐	☐	☐	☐	☐
8	V	errors or mistakes are seen as an opportunity for learning and improvement.	☐	☐	☐	☐	☐
9	V	dare all team members to be vulnerable.	☐	☐	☐	☐	☐
10	V	i feel safe.	☐	☐	☐	☐	☐
11	V	we encourage each other to learn new things.	☐	☐	☐	☐	☐
12	O	All team members are open to change.	☐	☐	☐	☐	☐
13	O	we pay attention to our computer skills	☐	☐	☐	☐	☐
14	C	all team members dare to speak up and raise everything	☐	☐	☐	☐	☐
15	C	all team members are open to each other, they listen and ask questions until it is clear what the other person thinks and wants	☐	☐	☐	☐	☐
16	C	team members do not get stuck in complaints and emotions, but come up with concrete proposals	☐	☐	☐	☐	☐

Sigra [33], freely translated from: Edmondson [34,35]
